# Vitamin D in Fibromyalgia: A Causative or Confounding Biological Interplay?

**DOI:** 10.3390/nu8060343

**Published:** 2016-06-04

**Authors:** Spyridon Karras, Eleni Rapti, Stauros Matsoukas, Kalliopi Kotsa

**Affiliations:** First Department of Internal Medicine, Division of Endocrinology and Metabolism, AHEPA Hospital, Thessaloniki 54636, Greece; raptieleni8@gmail.com (E.R.); mastparkour@hotmail.com (S.M.); kalmanthou@yahoo.gr (K.K.)

**Keywords:** chronic pain, 25-hydroxyvitamin D (25(OH)D), 1,25 dihydroxyvitamin D (1,25(OH)D3), vitamin D receptor (VDR), calcitriol, cholecalciferol, quality of life

## Abstract

Fibromyalgia (FM) is a chronic syndrome with an increasing prevalence, characterized by widespread musculoskeletal pain in combination with a variety of cognitive symptoms and fatigue. A plethora of scientific evidence that has accumulated during the last decades, resulted in a significant improvement of the understanding of the pathophysiology of the disease. However, current therapeutic approaches in patients with FM remains a multidimensional approach including patient education, behavioral therapy, exercise, pain management, and relief of chronic symptoms, rather than the use drug therapies, based on the mechanisms of disease development. Vitamin D, a fat-soluble vitamin derived mainly from skin synthesis through ultraviolet radiation, has been recognized to manifest a plethora of extraskeletal actions, apart from its fundamental role in skeletal and calcium homeostasis, including modulation of cell growth, neuromuscular actions, and potential anti-inflammatory properties. Recent findings indicate that hypovitaminosis D to be highly prevalent in patients with FM. Supplementation studies are limited so far, indicating potential beneficial effects on pain and severity of the disease, however specific recommendations are lacking. This review aims to summarize and critically appraise data regarding the pathophysiological interplay between vitamin D and FM, available results from observational and supplementation studies so far, with a clinical discourse on current knowledge gaps and future research agenda.

## 1. Introduction

Fibromyalgia (FM) is a chronic syndrome characterized by widespread musculoskeletal pain in combination with a variety of cognitive symptoms and fatigue, in the absence of another clinical disorder that could result in similar clinical manifestations [1,2,3]. It affects millions of people worldwide, with an impact on quality of life, resulting in limited social activity, isolation, and depression. A plethora of scientific evidence that has accumulated during the last decades resulting in a significant improvement towards the understanding of the pathophysiology of the disease. However, this progress has not been yet translated in the development of targeted drug therapies based on the phenomena characterizing the disease. Our current therapeutic approach in patients with FM remains an issue of multidimensional approach including patient education, behavioral therapy, exercise, pain management, and relief of chronic symptoms [4].

In this context, any biological molecules that could be implicated in the pathways that have been associated with the development of the disease could provide a potential target for novel therapeutic strategies. Vitamin D, a fat-soluble vitamin derived mainly from skin synthesis through ultraviolet (UVB) radiation, has been recognized to manifest a plethora of extraskeletal actions, apart from its fundamental role in skeletal and calcium homeostasis, including modulation of cell growth, neuromuscular actions, and a potential anti-inflammatory properties [5,6]. This narrative review aims to summarize and critically appraise data regarding the pathophysiological interplay of vitamin D and FM, available results from observational and supplementation studies so far, with a clinical discourse on current knowledge gaps and future research agenda. Although this article was not a systematic review, for this purpose we searched Pubmed.gov, using the terms “vitamin D, fibromyalgia, inomyalgia, supplementation” for related studies from 2000, by including all vitamin D supplementation trials in FM conducted so far with a special focus on the etiopathogenesis of the disease.

## 2. Fibromyalgia

### 2.1. Definition, Prevalence, and Diagnosis

According to the American College of Rheumatology (ACR), FM is a common chronic clinical condition characterized by widespread pain (CWP), accompanied by fatigue, sleep, memory, and mood disorders [1]. Diagnosis often is accompanied with a variety of general symptoms including irritable bowel syndrome, anxiety, and depression [1,2]. Fibromyalgia is the second most common disorder observed by rheumatologists, after osteoarthritis [1,2,3]. Women are more likely to develop FM, since 85%–90% of the affected populations are females [2,3,4]. Prevalence of FM varies considerably, according to the population group, as well as the methodology of the conducted survey [1,2,3,4,7]. It generally ranges between 0% and 3.7% [1,3,7] in men, and 0.75%–10.45% in women [1,3,8]. According to a prevalence study in France, FM was found to have the highest prevalence in 45–54 year-old age groups (2.5%–3.9% of the general population) and in 75–84 year-old age groups (3.9%–4.1% of the general population), while FM appeared to be uncommon in patients under 25 years old [3]. Another study in 1930 women with FM recorded the higher percentage (10.1%) in the 50–59 age group [9]. Low family income and low level of education have been positively correlated with the likehood of FM in previous studies [8,9,10].

In 1990, the ACR developed criteria for the FM diagnosis according to which FM was present, if at least 11 of 18 proposed trigger points were evident (Table 1), in combination with widespread musculoskeletal pain. However, there were several issues with the use of these criteria in daily clinical diagnosis of FM. In specific, the tender point count was performed rarely and/or incorrectly [11], making the diagnosis of FM primarily a symptom-based diagnosis. In addition, significant variation among FM patients in terms of disease severity was evident, in particular in assessing patients whose symptoms and tender points decreased, and as a result they failed to satisfy ACR 1990 classification definition [1]. ACR developed in 2010 new diagnostic criteria that did not use tender points, but they aimed to integrate severity scale-based symptoms based on characteristic features of FM, by developing a *Symptom Severity Scale* (SSS) [12], consisting of ordinal categorical variables describing general symptoms [11]. In addition, these criteria integrated the *Widespread Pain Index* (WPI), which is a 0–19 count of painful non-articular body regions. For the 2010 series of criteria, a diagnosis of FM can be made when levels of the *Widespread Pain Index (WPI)* and *Symptom Severity Scale (SSS)* are sufficiently high (WPI ≥ 7 and SSS ≥ 5 or WPI 3–6 and SSS ≥ 9). The WPI and the SSS is a 0–12 measure of symptom severity that includes fatigue, sleep, and cognitive problems [12] (Table 1). With this definition, a high level of symptoms is required in order to make FM diagnosis high likely, thus reflecting the severity of pain that the patient experiences. Those diagnostic criteria were not meant to replace the ACR classification criteria [1]. Instead, they were designed to improve diagnostic accuracy and avoid misclassification. The SS scale was strongly correlated with the WPI (*r* = 0.733) and the tender point count (*r* = 0.680) [12].

Recently, it was found that the underlying spectrum of severity that formed the basis for the 2010 criteria, could be visualized by adding together elements of the ACR 2010 criteria to form the polysymptomatic distress (PSD) scale [13]. The scale is obtained by summing the two components of the 2010 criteria, the WPI and SSS. The advantages of the criteria/PSD scale as a measure of severity are several. This scale is simple to use and to score, and is increasingly being used in patients with FM [14,15], providing a useful overall measure of FM severity. 

### 2.2. Who Should Be Screened for Fibromyalgia?

As described above diagnosis of FM is primarily based on patients self-reported duration and severity of symptoms. However, FM is also a diagnosis of exclusion. By taking into consideration that currently, no specific biochemical markers are considered as the gold standard for FM diagnosis, the clinician’s role is to consider and exclude other chronic clinical entities that may present with similar symptoms. Coexisting disorders such as rheumatoid arthritis, systemic lupus erythematosus, osteoarthritis, ankylosing spondylitis, or Lyme’s disease may further complicate appropriate diagnosis. It is very common that a complete hematological and biochemical check, thyroid and parathyroid, as well as immunologic/rheumatologic screening is required. Major risk factors for FM include the patient’s sex (FM is more prevalent in women than in men) [1,2,3], and patient’s family history (more likely to develop FM if a relative also has FM) [4]. 

Overall, screening for FM requires an individualized and holistic approach tailored on the patient’s medical history, appropriate clinical evaluation according current diagnostic criteria, targeted laboratory or imaging evaluation, and a high index of clinical suspicion.

### 2.3. How Do We “Understand” Pain in Fibromyalgia? 

Although the exact pathways that are implicated in the development of FM, remains to be elucidated, our current understanding of FM pathogenesis has increased substantially in recent years [16]. It has been hypothesized that a physical trauma, surgery, infection, or significant psychological stress could trigger events the pathogenesis of the disease [17]. Fibromyalgia may run in families and recent research suggests a strong genetic basis for FM. Recently, a possible relationship between the FMR1 premutation and FM has been pointed out [18,19]. 

Current research agenda however, focuses to central nervous system (CNS) homeostasis as the main regulatory mechanism implicated in the pathophysiology of FM [20]. Indeed, it has been hypothesized that patients with FM manifest CNS dysregulation in pain processing and an amplified response to stimuli that would not ordinarily be perceived in healthy individuals [20]*.* FM appears to be related with neurochemical imbalances and inflammatory pathways in the brain that result in amplification of painful sensation [18,19,20] with increased signaling in the ascending and decreased signaling in the descending neural pathways [21,22,23,24]. In addition, FM patients, also exhibit a decreased threshold of to a number of other sensory stimuli, including heat, cold, auditory, and electrical stimuli [21,22]. Previous pain threshold studies reported that patients with FM perceived pain stimuli at a lower threshold than healthy controls [22], while blood flow in brain areas associated with pain processing was augmented in FM patients, compared to healthy controls [23]. 

Homeostasis of pain neurotransmitters is also dysregulated in FM. An increase in excitatory neurotransmitter concentration (including substance P, nerve growth factor, and brain-derived neurotrophic factor) and amino acids (including glutamate) in the cerebrospinal fluid (CSF) and brain of FM patients compared to controls has been reported [20,21,24,25], indicating their contribution to hyperalgesia development in FM. CSF opioid levels are increased [26], whereas opioid receptor binding is decreased [27], resulting in an increase in baseline endogenous opioidergic activity. Daniel *et al.* [20] hypothesized that similar biophenomena in other brain regions, could result in mood disorders, sleep dysfunction, and fatigue frequently associated with FM. 

Other factors that may contribute to the increased pain perception in FM include abnormal autonomic function, hypothalamic-pituitary-adrenal axis abnormalities [28], neurogenic inflammation (glial cell activation) [29], and gray matter loss [30]. In the field of human studies, Bosma *et al.* [31], assessed the activity of brainstem and spinal cord through functional MRI after stimulating two groups of FM patients and controls, with stimuli with intensity adjusted to each participant's heat pain sensitivity to achieve moderate pain. Multiple areas in the brainstem (rostral ventromedial medulla and periaqueductal grey region) and spinal cord (dorsal horn) exhibited greater activity in controls, whereas an enhanced dorsal horn activity was demonstrated in FM patients. These findings were in accordance with the hypothesis of dysregulation of descending pain control mechanisms in FM patients.

In 2006, Carrillo *et al.* [32], recorded auditory-evoked potentials, (AEPs) elicited by tones of increasing intensity (60, 70, 80, 90, and 105 dB) in 27 female FM patients and 25 healthy controls. They reported that the most significant difference between patients and control subjects was at the highest stimulus intensity, and they concluded that the larger AEP amplitudes to the 105-dB tones suggests that defects in inhibitory mechanisms protecting against overstimulation may be a crucial factor in the pathogenesis of FM. At that basis, peripheral pain generators likely play some role, but probably through CNS activation, which becomes largely independent of peripheral nociceptive input [21]. Finally, a potential mechanism that could explain the acute nociceptive pain in FM, is a change in the activity of the nociceptors located in the affected anatomical structures (joints, tendons, and ligaments), which augments their sensitivity sensitive to normally painful stimuli (hyperalgesia), or normally non-painful stimuli (allodynia) [33].

## 3. Fibromyalgia and Vitamin D

### 3.1. Pathophysiology 

As described above, FM appears to be related with a neurotransmitter imbalance and upregulation of inflammatory pathways in CNS resulting in central amplification of peripheral pain signals [21,22,23,24]. Although available clinical results regarding the interface of chronic pain and hypovitaminosis D remain limited, a relative dearth of experimental and pathophysiological evidence demonstrate that vitamin D affects pain manifestation, thereby playing a role in the etiology and maintenance of chronic pain states and associated comorbidity [34,35,36]. Pain pathways associated with cortical, immunological, hormonal, and neuronal changes in chronic pain, are potentially also influenced by vitamin D levels [34]. 

In this context, recent research efforts focused on the potential future therapeutic implications of vitamin D, and its deficiency in the regulation of pain processing in CWP in FM, through complex central and peripheral interactions .The main functional background for this interplay is based on the presence of vitamin D receptor (VDR) and 1𝛼-hydroxylase [the enzyme that converts 25-hydroxyvitamin D (25(OH)D) by hydroxylation to the active 1,25 dihydroxyvitamin D (1,25(OH)_2_D3) in many areas of the human CNS. These include the prefrontal cortex, amygdala, raphe, substantia gelatinosa, cerebellum, hippocampus, cingulate gyrus, substantia nigra, thalamus, and hypothalamus [37,38,39,40,41]. Both the receptor and the enzyme have been demonstrated in neuronal and glial cells as well [37]. In the rat model, vitamin D binding protein (VDBP) has been found in axonal projections in the lateral hypothalamus [38]. The presence of VDR, 1𝛼-hydroxylase, and VDBP in the hypothalamus is suggested as the mechanism by which vitamin D deficiency is implicated in the pathophysiology of CWP in FM [40]. 

Vitamin D seems to play an important role in brain development, to have a potential neuronal regulatory action, to promote different nerve growth factors and also exerts neuroprotective effects [34]. Vitamin D can modulate neuronal excitability similar to that of other neuroactive steroids. This includes spontaneous regular firing, action potential duration, intrinsic excitability, and sensitivity to neurotransmitters as well as to neurotransmitter receptors such as γ-aminobutyric acid (GABA) receptor and *N*-methyl-d-aspartate (NMDA) receptor [41]. GHB is an endogenous compound, synthesized from γ-aminobutyric acid (GABA) which, in a form of sodium salt (SXB), demonstrated significant efficacy in FM but also a high rate of adverse events [42].

In addition, vitamin D seems to be involved in the production of Glial Cell line-Derived Neural Growth Factor (GDNF), a neuropeptide associated with protective actions regarding the growth and maintenance of sympathetic and sensoral neurons in CNS [43]. Primary glial cells that were exposed to 1,25(OH)_2_D3 revealed a marked increase in secretion of GDNF, directly correlated to the duration of the treatment [44]. It has to be noted that previous results indicated significantly lower CSF concentrations of GDNF in FM patients compared to controls [45], indicating a potential indirect beneficial effect of vitamin D modulation. As a steroid, vitamin D and its metabolites also modulate different brain neurotransmitters (acetylcholine, dopamine, and serotonin) as well [34], which are modulated by the neurotrophic activity of 1,25(OH)_2_D [34]. Dopamine system in FM has been characterized previously through positron emission tomography (PET) with [18F] fallypride, to assess changes in dopaminergic activity during memory tasks [46]. In FM, abnormal dopamine function was associated with a dysregulation of pain processing in FM. These results indicate an additional theoretical role of vitamin D deficiency in the pathogenesis of FM.

Vitamin D is also known to affect a number of inflammatory pathways associated with the development and persistence of chronic pain. Vitamin D upregulates transforming growth factor beta 1 (TGF-β1) and interleukin-4 (IL-4) found in astrocytes and microglia [47]. TGF-β1 suppresses the activity of various cytokines, namely, interferon-γ, TNF-α, and various T cells such as interleukin-1 (IL-1) and interleukin-2 (IL-2) [47]. On the other hand, FM is associated with disturbance in immune regulation by an increased concentration of TNF-α, which has been convincingly implicated at both peripheral and central levels of sensitization [48]. 

Vitamin D also seems to be involved in the production of nitric oxide (NO) by restraining the synthesis nitric oxide synthase (NOS) through which NO is produced. NO is a bioproduct and an important biological regulator which effects neurotransmission and vasodilation [47]. It is produced by phagocytes as part of human immune response and the inhibition of NOS by vitamin D could provide a rational therapeutic approach, since in a previous study NOS activity has been found significantly higher in FM patients compared to controls and NOS levels correlated with chest pain and dyspnea [48]. However, it should be noted that ultraviolet exposure (UV) also increases NO and this effect might result from UV exposure, rather than vitamin D per se [49,50]. 

At a peripheral level, chronic persistent pain has been also associated with myopathy, and musculoskeletal pain. Vitamin D seems to possess anti-inflammatory properties which may alter peripheral pain sensitivity [37,39,51,52]. Vitamin D seems to increase muscle strength through nuclear receptors in muscle tissue. In humans, vitamin D hypovitaminosis results in myopathy especially in the size and number of Type II muscle fibers and fatty infiltration of skeletal muscles [6,51]. Recent evidence suggests that patients with osteomalacia suffer from muscle atrophy and decreased muscle strength [51]. In addition, a recent study investigated the correlation of central hypersensitivity to pain in patients diagnosed with chronic pain (47% FM by ACR criteria) and the results showed that 75% of the group were vitamin D deficient (<50 mmol/L) and the degree of 25(OH)D deficiency corresponded to the degree of pain sensitivity [52]. 

Results regarding the role of hypovitaminosis D on bone mineral density (BMD) in FM patients are conflicting. Olama *et al.*, [53], assessed vitamin D concentrations and BMD value in patients with FM (*n =* 50) and 50 age-matched healthy controls. Patients with FM had significantly lower serum 25-OHD than controls (15.1 ± 6.1 and 18.8 ± 5.4 ng/mL, respectively, *p* = 0.0018) and significantly lower BMD in the lumbar spine, compared with controls (*p* = 0.0012.) Serum 25(OH)D concentrations were inversely correlated with visual analogue scale (VAS) of pain (*p* = 0.016), Beck score for depression (*p* = 0.020) and BMD at lumbar spine (*p* = 0.012). This study confirmed that hypovitaminosis D is a risk factor for low lumbar BMD in FM and correlated with pain severity [53]. However, recent findings did not confirm these results. A recent study also assessed BMD in 205 patients with FM and 205 healthy controls using dual absoptiometry. No differences in BMD were evident between the two groups [54].

Tague *et al.* [55], in a recent animal study demonstrated that a vitamin D deficient diet contributed to deep muscle hypersensitivity and a balance deficit which occurred even before deterioration of bone health. The study also indicated that vitamin D deficiency can selectively cause hyperinnervation of skeletal muscles which is correlated to muscle pain and heightened sensitivity to pain stimuli. Based on the above phenomena, profound vitamin D deficiency could result in alterations of muscle physiology and mitochondrial defects and result in a variety of muscle disorders like myalgia, muscle tenderness, and muscle weakness, symptoms which are common in FM [1,2,3,4].

**Figure 1:**
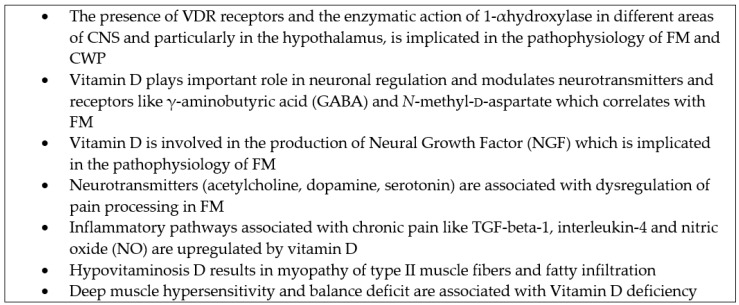
Hypothetical pathophysiological mechanisms of vitamin D and FM interplay. Abbreviations: VDR: Vitamin D receptor; FM: Fibromyalgia; CNS: Central nervous system; CWP: Chronic widespread pain.

Overall, although a cause and effect relationship has not been proven yet, available evidence indicates, that vitamin D is a vital bioregulator of pain pathways involved in FM pathogenesis (Figure 1). However, significant issues regarding the optimal concentrations of vitamin D attained in CNS in order to exert its pleiotropic actions and to which extent these actions are influenced by VDR polymorphisms or serum vitamin D concentrations remain unanswered.

Hypovitaminosis D may be a risk factor for FM and a way of worsening the symptoms through central and peripheral pathways. The exact mechanisms however, by which vitamin D may be related with FM remain unclear [56,57]. 

### 3.2. Data from Observational Studies and Meta-Analyses

During the last decade, several observational studies attempted to investigate the association of hypovitaminosis D and symptoms of CWP and FM. Initial reports, although included small samples, demonstrated that hypovitaminosis D is frequently seen in FM [58,59].

Plotnikoff and Quigley reported the prevalence of vitamin D deficiency in patients with nonspecific musculoskeletal pain. A total of 100% had deficient concentrations of vitamin D (<or =20 ng/mL). Of all patients, 93% (140/150) had deficient levels of vitamin D (mean, 12.08 ng/mL; 95% confidence interval, 11.18–12.99 ng/mL). Of major interest, males and females were equally deficient, whereas 28% of the patients with non-specific musculoskeletal pain had vitamin D concentrations lower than 8 ng/mL [39]. In addition a large male European cohort study [60], which included 2313 men, with an average age of 58.8 years, aimed to determine the relationship between low vitamin D concentrations and the risk of developing CWP for a mean duration of follow up at 4.3 years. Results revealed a highest risk of developing CWP for participants being in the lowest quintile (<15.6 ng/mL) compared to those in upper quintile of 25(OH)D (≥36.3 ng/mL), after adjustment for age and centre, physical performance and number of comorbidities (Odds Ratio (OR) = 1.93; 95% CI = 1.0–3.6). Further adjustment for BMI (OR = 1.67; 95% CI = 0.93–3.02) or depression (OR = 1.77; 95% CI = 0.98–3.21), however rendered the association non-significant.

On the other hand, Tandeter *et al.*, found no statistically significant differences in premenopause were evident, between FM patients (*n =* 68) and healthy controls (*n =* 82) in 25(OH)D concentrations groups regardless of the cutoff level used [61]. 

Hypovitaminosis D has been also correlated with severity of FM symptoms. In a previous study in 75 Caucasian patients who fulfilled ACR criteria for FM, serum vitamin D concentrations were evaluated. In addition, participants completed an adjusted fibromyalgia impact questionnaire (FIQ) and a Hospital Anxiety Depression Score (HADS). Hypovitaminosis D was evident in 13.3% of the patients, while 56.0% had insufficient and 30.7% had normal concentrations. Patients with vitamin D deficiency (<25 nmol/L) had higher HADS (median, IQR, 31.0 (23.8–36.8)) than patients with insufficient levels (25–50 nmol/L; HADS 22.5 (17.0–26.0)) or than patients with normal levels (50 nmol/L or greater; HADS 23.5 (19.0–27.5); *p* < 0.05)) [62].

However, not all previous evidence report similar findings. De Rezende Pena and colleagues [63] in a cross sectional study, evaluated the differences in 25(OH)D concentrations, between 87 FM patients and 92 normal subjects and reported no statistical differences and no association between pain scales and tertiles of 25(OH)D. Previous studies manifest great variation in ethnical, cultural, and dietary habits, factors that have major impact on vitamin D status [64,65]. Moreover, data regarding previous vitamin D supplementation in study participants are not available in most studies [60,61,62,63]. Additional parameters of heterogeneity, including differences in criteria used for vitamin D deficiency, small study samples, and lack of control groups are also evident [64,65]. 

However, these results were gradually incorporated into systematic analyses on the field. 

A recent meta-analysis of observational studies, included 12 studies (eight of which the patients were diagnosed with FM and the rest with CWP), comprising of 1854 patients and 7850 controls [66]. The patient group showed a significantly higher risk of hypovitaminosis D than the control group (OR, 1.63; 95% CI, 1.20–2.23). The association was slightly attenuated after adjusting confounders, with a pooled adjusted OR of 1.41 (95% CI, 1.00–2.00). There was an increase in ORs of hypovitaminosis D using a lower diagnostic value of serum vitamin D (8 and 10 ng/mL). The subgroup analysis according to gender and definition of CWP did not reveal significant between-group differences. It was suggested that values of vitamin D <10 ng/mL may be able to better differentiate the affected population from the control group. Furthermore, the results of this meta- analysis implied that hypovitaminosis D in these patients could depend on factors such as sun exposure, seasonal variation, body mass index, and physical activity but further research is required to investigate these effects [66]. 

### 3.3. Supplementation Studies

Only a few studies have tested the theory that supplementation with vitamin D can be beneficial in managing the symptoms of FM (Table 2). In 2009, Badsha *et al.* [67] treated by administrating high doses of vitamin D, patients diagnosed with FM and/or nonspecific muscle pain and hypovitaminosis D. Depending on the measured vitamin D status patients were assigned to receive vitamin D either as a single dose intramuscular (i.m.) injection or oral weekly supplementation for eight weeks. At follow up, patients demonstrated clinical improvement in their symptoms. However attained vitamin D concentrations were not reported and clinical improvement was not defined according ACR criteria.

In another study by Harari *et al.* [68], 33 Norwegian patients with a diagnosis of low back pain or FM were exposed in a treatment protocol which included daily sun exposure and bathing in Dead Sea water for a three week period. Of major interest, patients baseline 25(OH)D concentrations in the FM group were recorded at the sufficiency range (75.5 ± 28.1 nmol/L), whereas a significant increase (88.8 ± 23.8 nmol/L) after sun exposure was evident, which was also associated with attenuation of pain related symptoms. However, baseline concentrations could be attributed to vitamin D supplementation prior to inclusion to the study, especially considering the country of residence of participants, where vitamin D fortification of dairy products is implemented in public health policy. In 2011, Matthana *et al.* [69], in a prospective cohort study, included 61 vitamin D deficient women from Saudi Arabia suffering from FM and treated them with 50,000 IU ergocalciferol once weekly. 42 of these demonstrated improvement in FM symptoms when 25(OH)D concentrations attained sufficiency (>30 ng/mL) and further improvement of symptom scores when 25(OH)D was >50 ng/mL. In a similar study [70], 30 females with FM from Saudi Arabia and hypovitaminosis D (4.76 ± 1.46 ng/mL) were included in a supplementation study with high doses of vitamin D (600.000 IU i.m. single dose or orally 50,000 IU/week for 8 weeks). Patients were re-evaluated according ACR criteria at follow up one month after treatment with injection, or two months after oral therapy. 

Treated patients had significant clinical improvement on multiple aspects of new clinical FM diagnostic criteria [12] apart from cognitive symptoms. However, in addition to the absence of a control group, attained concentrations of 25(OH)D were not available after supplementation. 

On the other hand, in 2008 Warner *et al.* [71] recruited 50 patients of fair skin with diffuse pain and 25(OH)D concentrations ≤20 ng/mL and randomized to receive supplementation with 50,000 IU ergocalciferol or placebo once weekly for a three-month period. Outcomes assessed were pain measured by visual analog scale (VAS) and functional pain score (FPS). Mean 25(OH)D concentrations were not different between groups. Vitamin D supplementation in deficient participants resulted in no improvement in VAS and FPS compared to placebo. In a similar small randomized control trial, Wepner *et al.* [72] recruited 30 women with FM and vitamin D concentrations ≤32 ng/mL. The treatment group received oral cholecalciferol for 20 weeks and the patients were scheduled to be re-evaluated after 44 weeks. In the treatment group, a significant reduction in symptoms was noted. In specific, VAS and pain perception significantly improved and this effect was also correlated with scores on the physical role functioning scale of the Short Form Health Survey 36.

Overall, in the field of vitamin D supplementation as a potential therapeutic strategy in FM, several limitations in the available studies complicate arrival at safe clinical conclusions. Most previous studies did not include control groups, or included samples were small with a significant variation in population and climate parameters (seasonal variation, weight, and diet). 

In addition, the discrepancy of available trials could be also the result of the vitamin D supplements used in each trial. It has been suggested [73], that vitamin D_2_ (ergocalciferol) is less potent in attaining physiological concentrations of 25(OH)D compared to vitamin D_3_ (cholecalciferol) and previous reviews reported that in the absence of concomitant use of calcium supplements, compared with vitamin D_3_, vitamin D_2_ was associated with a significantly lower overall increase in serum 25-hydroxyvitamin D concentration [74]. 

Of major interest, in a recent meta-analysis of observational and supplementation trials, subgroup analyses showed that vitamin D_2_ supplementation increased the aggregate risk of mortality in trials that had a short average intervention period and used low average doses of supplementation [75]. 

An ideal vitamin D supplementation trial would use a reference population, with different baseline vitamin D status, aiming at attaining sufficient serum 25(OH)D concentrations, in order to establish a “supplementation and result” relationship [76]. Nevertheless, this outcome could be significantly affected by the regimen and dose of vitamin D used in each study. This issue becomes of outmost importance in the field of vitamin D and FM, since some studies used D2 as a supplement, affecting the studied outcomes. Since even a large bolus of 50,000 or 100,000 IU of vitamin D would rapidly (in a few days) be absorbed and undetectable from the serum [77], it has to be noted that several supplementation trials used this type of bolus administration, with a potential effect on their outcomes [67,69,70,71]. 

In this context, the duration of supplementation could also play a role in maintaining adequate vitamin D concentrations. Although the optimal dosing and duration for specific outcomes remains to be defined, by supplying constant doses of vitamin D for three or four months, a steady state will be attained [78]. This is not the case in bolus regimens with monthly or weekly patterns of supplementation. Nevertheless, the supplementation with vitamin D seemed to be effective in ameliorating some of the symptoms that these patients experienced and should be warranted in these FM patients for preserving bone health until beneficial effects on pain perception are confirmed by future large-scale studies.

## 4. Future Perspectives of Vitamin D Clinical Trials in Fibromyalgia

FM is a chronic condition with an increasing prevalence worldwide. Although pain perception could be considered a universal index, individual parameters could affect daily clinical interpretation of related symptoms. On the other hand, as vitamin D is generated by an environmental factor (sunshine exposure), it can also be affected by local geographical factors [79]. These observations indicate that vitamin D could vary significantly within a country, particularly in areas with a wide range of latitude gradient. Moreover, vitamin D status of immigrant populations in Europe was poor compared with that of the indigenous European populations [80], indicating that social and cultural habits are different as well. It becomes evident that appropriate interpretation of vitamin D status before and after supplementation in FM should take into account specific geographical characteristics, such as latitude, UVB radiation, and microclimate, as well as the specific social and dietary habits.

Baseline 25(OH)D concentrations in conjunction with ethnicity and individual response to solar UVB, according to skin phototype and racial variances in alleles of vitamin D-binding protein (VDBP) could minimize heterogeneity among future observational and supplementation studies in FM [78]. In the case of vitamin D supplementation studies, it has been hypothesized that supplementing populations with lower baseline concentrations could give better results. This parameter could affect results of studies where vitamin D supplementation was used. We failed to achieve optimal concentrations of 25(OH)D particularly in countries with lower 25(OH)D concentrations in general, such as Middle Eastern countries [67,68,69,70]. This approach, however, should be combined with appropriate supplementation regimens, in order to achieve sufficient 25(OH)D levels in these populations. Currently, vitamin D should be recommended in all FM patients at high risk of developing hypovitaminosis D like insufficient sun exposure, inadequate dietary intake, and obesity [35].

## 5. Conclusions

Previous clinical results indicated hypovitaminosis D to be highly prevalent in patients with FM and some reported an improvement in the clinical scale symptoms after vitamin D supplementation. 

However, the involvement of vitamin D in the pathogenesis of FM, as well as its potential therapeutic role after supplementation, remains to be proven and fully elucidated**.** Future appropriately designed supplementation trials, tailored to specific populations and targeting specific cut off values, might offer a new therapeutic approach on the field. Currently, scientific evidence supports that vitamin D supplementation cannot be routinely recommended in FM in daily clinical practice. However, vitamin D supplementation in cases with a high-risk of developing vitamin D deficiency or documented profound hypovitaminosis D is recommended. 

## Figures and Tables

**Table 1:** ACR criteria needed for fibromyalgia diagnosis and classification.

Older Criteria	Widespread Pain, Both Halves of the Body, ≥3 Consecutive Months ≥11 of the Tender Points (see Text)
Current Criteria (as referred in ACR official website)	Pain and symptoms over the past week, based on the total number of: painful areas out of 19 parts of the body PLUS level of severity of these symptoms: a. Fatigue; b. Waking unrefreshed; c. Cognitive (memory or thought) problems PLUS number of other general physical symptoms. Symptoms lasting at least three months at a similar level. No other health problem that would explain the pain and other symptoms

ACR: American College of Rheumatology.

**Table 2:** Available vitamin D supplementation studies in FM.

Study and Year of Publication	Population	Methodology	Outcome	Comment
Badsha *et al.*, 2009 [67]	FM Patients (*n =* 139) or muscle pain	*N =* 50 i.m. 600.000 IU Vit D3 (<15 ng/dL), single dose	Follow up 1–2 months	Serum 25(OH)D after supplementation not reported
		*N =* 20 p.o. 50.000 IU Vit D3/week for 8 weeks (<15 ng/dL)	90% of patients reported clinical improvement		
	95% female (age 40 ± 11.5)			
		*N =* p.o. 1 mg alphacalcidiol (16–20 ng/dL)		
	Middle East			
Harari *et al.*, 2011 [68]	Patients (*n =* 60) age 62.8 ± 11.6	Daily sun exposure, balneotherapy	Group 1: 25(OH)D: 88.8 ± 23.8 nmol/L (35.5 ± 9.5 ng/mL)	Data on vitamin D supplementation prior to inclusion to the study not reported
	Females 47, males 13			
	Group 1 (*n =* 33) FM 25(OH)D: 75.5 ± 28.1 nmol/L (30.2 ± 11.2 ng/mL)		Group 2: 25(OH)D: 84.7 ± 22,4 nmol/L (33.9 ± 8.81 ng/mL)	
	Group 2 R.A.(*n =* 16), 25(OH)D: 63.6 ± 27.8 nmol/L (25.4 ± 11.1 ng/mL)			
			Group 3: 25(OH)D :97.3 ± 23 nmol/L (38.9 ± 9.21 ng/mL)	
	Group 3 OA *n =* 11, 25(OH)D: 71.8 ± 18.5 nmol/L (28.7 ± 7.4 ng/mL)		VAS levels improved significantly from 4.88 ± 1.63 to 7.26 ± 1.46	
Matthana *et al.*, 2011 [69]	FM female patients (*n =* 100) *n =* 61 Vit D deficient Saudi Arabia	Patients with vit. D deficiency treated with vit.D2 50.000 IU/week until their blood level of 25(OH)D exceeded 50 ng/mL	42 reported significant clinical improvement (FIQR) (25(OH)D ≥30 ng/mL)	No controls included
Abokrysha *et al.*, 2012 [70]	FM patients (*n =* 30) age 34.56 ± 8.1	Patients received either i.m 600.000 IU Vit D3	Patients demonstrated significant clinical improvement (ACR criteria)	No controls included concentrations of 25(OH)D were not available after supplementation
	Vit D 4.76 ± 1.46 ng/mL Saudi Arabia	Single dose or p.o. 50.000 IU Vit D3/week (8 weeks)		
Warner *et al.*, 2008 [71]	FM female patients (*n =* 50) (Diffuse pain) Vit D ≤ 20 ng/mL	*n =* 25 placebo (age 56.7 ± 11.3) *n =* 25 vit.D2 (50.000 IU/week) for 3 months (age 58 ± 7.3)	Vit D in the active treatment group higher than placebo 31.2 ± 6.1 ng/mL *vs.* 19.3 ± 6.5 ng/mL No improvement in FPS and pain on VAS	Data on vitamin D supplementation prior to inclusion to the study not reported
Wepner *et al.*, 2014 [72]	FM female patients(*n =* 30) Randomized in treatment group (TG) and control group (CG) 25(OH)D < 32 ng/mL	Randomized placebo controlled trial 20 weeks oral vit.D3 (First evaluation) Re-evaluation after 24 weeks without supplementation	Marked reduction in pain in TG (VAS) Optimization of 25(OH)D in FM had a positive effect on the perception on SFHS 36	Data on vitamin D supplementation prior to inclusion to the study not reported

**Abbreviations:** FM: Fibromyalgia; Vit D_3_: Vitamin D_3_; R.A: Rheumatoid arthritis; OA: Osteoarthritis; VAS: Visual analog scale; FIQR: Fibromyalgia impact questionnaire; ACR: American College of Rheumatology; SFHS 36: Short form Health Survey; FPS: Functional pain score.
